# Modeling for Fidelity: Virtual Mentorship by Scientists Fosters Teacher Self-Efficacy and Promotes Implementation of Novel High School Biomedical Curricula

**DOI:** 10.1371/journal.pone.0114929

**Published:** 2014-12-31

**Authors:** Katherine Malanson, Berri Jacque, Russell Faux, Karina F. Meiri

**Affiliations:** 1 Department of Developmental, Chemical and Molecular Biology and Center for Translational Science Education, Tufts University School of Medicine, Boston, Massachusetts, United States of America; 2 Davis Square Research Associates, Somerville, Massachusetts, United States of America; University of Westminster, United Kingdom

## Abstract

This small-scale comparison case study evaluates the impact of an innovative approach to teacher professional development designed to promote implementation of a novel cutting edge high school neurological disorders curriculum. ‘Modeling for Fidelity’ (MFF) centers on an extended mentor relationship between teachers and biomedical scientists carried out in a virtual format in conjunction with extensive online educative materials. Four teachers from different diverse high schools in Massachusetts and Ohio who experienced MFF contextualized to a 6-week Neurological Disorders curriculum with the same science mentor were compared to a teacher who had experienced an intensive in-person professional development contextualized to the same curriculum with the same mentor. Fidelity of implementation was measured directly using an established metric and indirectly via student performance. The results show that teachers valued MFF, particularly the mentor relationship and were able to use it effectively to ensure critical components of the learning objectives were preserved. Moreover their students performed equivalently to those whose teacher had experienced intensive in-person professional development. Participants in all school settings demonstrated large (Cohen's d>2.0) and significant (p<0.0001 per-post) changes in conceptual knowledge as well as self-efficacy towards learning about neurological disorders (Cohen's d>1.5, p<0.0001 pre-post). The data demonstrates that the virtual mentorship format in conjunction with extensive online educative materials is an effective method of developing extended interactions between biomedical scientists and teachers that are scalable and not geographically constrained, facilitating teacher implementation of novel cutting-edge curricula.

## Introduction

The poor performance of high school students against international benchmarks raises concerns about the current state of U.S. science education [1]–[4]. One particular challenge undoubtedly affecting performance is the low level of student engagement in science [5]–[10]. One way of increasing engagement is to teach students content they value and find relevant to their lives: When students value what they are learning their motivation and achievement increases [11]–[13]. Students particularly value learning about health and disease, and high school biology curricula focused on health and disease stimulates interest and engagement in science, and concomitantly, academic performance [14]. This type of curricula may also foster students' health literacy capacities [15].

One major challenge to bringing life-relevant health science to the high school biology classroom is the requirement for in-depth knowledge about the biomedical sciences, topics few teachers are familiar with. We have the addressed this challenge via The Great Diseases Project, a learning collaborative involving biomedical content specialists and teacher leaders from a number of school districts [14]. The partnership has developed an innovative high school health science curriculum that provides a health-based context for learning biology. Targeted to Biology II (elective biology), ‘The Great Diseases’ has a modular framework focused on four globally significant diseases: Neurological Disorders, Infectious Diseases, Metabolic Disease and Cancer. It is inquiry-based and aligned with the most recent science education standards that relate to authentic science practices and the three dimensions of the NGSS (See Fig. A in S1 File) [16]. The GD is comprehensive (each disease module takes about 6 weeks of in-class time) and has been designed to build scientific understanding and also foster health literacy by teaching students how to critically evaluate scientific health claims and make connections between current developments in biomedical science and their health.

Successful implementation of novel curricula requires adequate teacher support and teacher self-efficacy [17]–[20]. Given their limited exposure to biomedical topics, few teachers have the self-efficacy to teach health science topics even when a relevant curriculum is made available. One practice highly correlated with improving self-efficacy and quality of implementation is professional development (PD) [21]–[25]. Evidence indicates that the most effective PD focuses on providing content contextualized to pedagogical content knowledge within a framework of active learning [17], [26], [27]. This means that embedding biomedical science content for teachers in the pedagogical context it will be used in a health science curriculum may be an effective way to teach content while building self-efficacy for implementation. Additionally, interactions with a mentor that are prolonged both in the total number of contact hours and duration of the experience leads to more extensive changes in teacher practices than the traditional model of time-limited workshops [26]. PD designed to incorporate these best practices positively impacted teachers' self-reported increase in knowledge, skills and change in practice [17]. It should be noted however that these results have not yet been linked to changes in student performance. Despite this research, limited time (day- or week-long) workshops have persisted as a preferred venue for PD [21]–[23], reflecting the dual challenges of sustaining long-term interactions and of addressing the individual needs of teachers. Some PD opportunities have been attempting to circumvent these challenges by moving to virtual or online platforms.

Several advantages of online PD are immediately evident: First, it is inherently asynchronous, accommodating individual teacher's schedules and thereby potentially attracting a larger audience. Second, it can incorporate synchronous elements that provide an opportunity for support and reflection. Third, it can be personalized to allow teachers to focus on material most relevant for their own classrooms. Finally, geographically remote teachers can access these resources [28]. As a result some models of online PD are more scalable than programs limited to in-person interactions. Nonetheless it also presents clear challenges: The best practices of online PD remain relatively undefined [28], and converting the evidence-based best practices established for in-person PD, namely active learning approaches and prolonged duration of mentor-focused interactions [17], [24], to an online approach is an active area of research [28]–[30]. Additionally, how will an online environment foster trust and collegiality that is instrumental in sharing knowledge and offering support without in-person interactions?

As part of the Great Diseases project we had offered graduate-level summer in-person PD courses that provided local teachers with contextualized content learning and prolonged mentor-focused support as they piloted curriculum modules in their classrooms [14]. However our goal was to make the Great Diseases curriculum accessible to teachers geographically distant from Tufts University in Boston, where the program is housed, while still providing opportunities for the synchronous interactions critical for successful implementation. Recognizing that biology curricula often fail to support teachers as they translate complex concepts for novice learners [31] we therefore created an innovative PD program called ‘*Modeling for Fidelity*’ (MFF) that synthesizes the best practices of in-person and virtual PD through a combination of asynchronous and synchronous learning, scientist mentoring and pedagogical support. MFF supports teachers as they implement the Great Diseases curriculum in their classrooms. In contrast to more traditional PD approaches that emphasize exploration of teaching methods [17], [26], [32] our program focuses on enhancing content knowledge contextualized to the curriculum and its learning objectives. MFF uses a hybrid format in which an online platform is extended to include personalized, though still virtual, interactions between the teacher and mentor and is thereby able to incorporate the evidence-based “best practices” for in-person PD [17], [26]. It has three objectives: *First*, it aims to increase teachers' content knowledge of the biomedical science underlying the Great Diseases topics covered by the curriculum. *Second*, it aims to contextualize this learning to the pedagogical practices used in the curriculum. *Third*, it aims to establish prolonged mentor relationships between teachers and scientist mentors to facilitate implementation.

### Purpose of this study

Here, we have used the neurological disorders (ND) module of the Great Diseases curriculum as a vehicle to compare a traditional in-person PD that reflects best practices – we call this comparison PD approach ‘Gold Standard’ – with our virtual MFF PD approach. We compared effects on teachers directly, using Fidelity of Implementation metrics (FOI) metrics modified from a previously established suite of instruments [33] and indirectly by measuring student achievement with two objective metrics, one focused on conceptual knowledge inventory and problem solving critical for health science literacy, the second focused on self-efficacy in learning about the topics [14]. The results show that teachers' abilities to implement the critical instructional components of the module, as reflected by the significant gains in student achievement on all matrices, occurred regardless of the form of PD the teachers had experienced, indicating that the MFF PD approach can be used to train teachers to implement novel curricula that use a variety of practices with unfamiliar content.

## Methods

### Study Design

This study compared enactments of the ND module in four self-selected schools with a diverse range of students. These include: In Massachusetts, an urban college-preparatory public high school, an urban general public high school with a high proportion of minority English language learners (Hispanic) and a suburban public high school with a wide range of student abilities; in Ohio, a regional public STEM high school with a diverse demographic (Table A in S1 File). The four teachers included in this case study represent a wide range of experience; a novice first year teacher; a teacher with extensive neuroscience experience; a teacher whose background was in chemistry rather than life science; and an expert teacher who had been recognized as State Biology Teacher of the Year. The teacher from the urban college-preparatory public high school received ‘gold standard’ traditional PD, defined below, was compared with the other three teachers who participated in the MFF virtual PD program. The same science partner worked with all of the teachers, but never met the MFF teachers in person. The outcomes of curriculum implementation the 3 schools whose teachers received virtual PD were then compared with the outcomes in the school whose teacher received ‘gold standard PD′. In addition one teacher, from the urban general public high school, had previously received ‘gold standard’ PD for the Infectious Disease module, and the outcomes of both modules on the same set of students were also compared directly.

### Data collection and measures

The study was carried out between 2011–2013 with 212 11–12^th^ grade students who experienced the ND module as part of an elective second level Biology course, an elective Physiology course, or an elective Psychology course. No students refused to participate in the study, however only 175 students (82%) completed both the pre- and post-conceptual knowledge inventory and only 147 (69%) completed the attitudinal surveys and are included in the analyses (Table B in S1 File). Participating students did the pre- and post-tests during the first and last lessons respectively. They were told that the pretest was a way for them to explain their initial understanding, and that their grades would not be affected by their performance. Both questions and answers were returned to the university researchers for grading. Retrospective pre-post surveys were done online as homework.

### Ethics statement

The study and surveys were approved by the Institutional Review Board of Tufts University School of Medicine under protocol #9049 as follows: Teachers signed institutionally approved forms to signify their informed consent to participate in the study and to publish the results. Student pre-and post-tests were given as part of their normal classwork and were de-identified. Student online surveys were anonymous and voluntary. Thus, the IRB considered that the study was exempt from the necessity to obtain written consent for students.

### Fidelity of implementation

Teachers were given questions designed to probe whether critical instructional and structural components i.e. our intentions about how the material should be used, were addressed in each lesson: They filled in online questionnaires about each lesson and were also questioned about the lessons by the science partner during the synchronous real-time reflection protocol (Table C in S1 File). A second online questionnaire asked teachers to reflect on the PD process itself and consists of six open-ended questions. Teachers were encouraged to complete it upon conclusion of the module in their classrooms (Table D in S1 File).

#### Student pre- and posttest ND conceptual knowledge and problem solving inventory

The conceptual knowledge inventory pre-post tests were designed to measure both content understanding and problem-solving abilities related to the crosscutting concepts in the ND module and health claims evaluation. They consisted of 10 multiple-choice items, 2 short response items and a clinical case study with 5 short response questions. Multiple-choice questions required recalling and comprehending information, while short answer questions and the case study required open-ended responses that asked students to draw conclusions based on evidence, create hypotheses and extrapolate from concepts to explain new phenomena. Questions were designed to be challenging in order to avoid a plateau effect and multiple questions per crosscutting concept established reliability. The assessment was reliable as measured by Cronbach's α (0.81).

#### Student self-reported changes in attitude and self-efficacy

We measured attitude and self-efficacy of students towards learning about ND material with an online survey consisting of 12 questions regarding attitude and 9 questions for self-efficacy, all questions used a six-point Likert-type response scale, (1 =  low, 6 =  high). The retrospective pre-test model, in which respondents are asked to recall a previous state and then report on their current state, is effective at avoiding Type II error, which was of concern in this study because of students' lack of prior exposure to health science learning [34], [35]. However, the retrospective method can produce inflated effect sizes (calculated using Cohen's ‘d’) if respondents deem it more socially desirable to over-estimate as they report gains [34]–[37]. We attempted to mitigate this effect by collecting responses online, anonymously. The assessments were reliable as measured by Cronbach's α (0.92).

#### Data analysis

Teacher data about fidelity of implementation is represented qualitatively due to the small sample size. Students' ability to use test-taking strategies to answer multiple-choice questions was minimized by requiring multiple correct answers to be identified and penalizing incorrect choices. Grading rubrics for short answer questions and the case study were established by ND content specialists, and each pre- and post-test was graded by two evaluators, whose scores were averaged. Scoring between the evaluators had a correlation coefficient of 0.94. The gains in student conceptual knowledge inventories were calculated post- pre-test, significance was detected with paired t-tests and effect size was analyzed using Cohen's d. Effect size is defined as small (ES ≥0.2, ≤0.5), medium (ES ≥0.5, ≤0.8) and large (ES ≥0.8, [38]). The Cronbach's alpha for each assessment was well above the 0.70 threshold generally considered reliable. Although these instruments are awaiting additional subject participation for validation, the magnitude of student gains means that the main risk of using a non-validated instrument, i.e. false negatives, is not of significant concern here.

### The Great Diseases (GD) curriculum

GD comprises a rich mosaic of pedagogy embedded in a constructivist framework. It uses Socratic and case-based discussions to continually challenge students to think critically, evaluate evidence and express their ideas and a multiplicity of pedagogical strategies to promote accessibility of the curriculum to different types of learners [39]. GD aligns with current science education standards [40]. The Neurological Disorders (ND) module was built around a framework of five questions selected by a team of ND content specialists from Tufts Medical School to represent the critical knowledge necessary for understanding ND and facilitating future learning about ND topics (Table E in S1 File). Each question in the framework comprises a unit of 5–7 individual lessons, totaling 6 weeks of class time. Each lesson provides a solid foundation of cutting-edge information readily transferable to real-world health situations.

The design partnership that originally developed the GD curriculum included teachers from the urban college preparatory school [14]. Because of this, the teacher from that school who implemented the ND module had received ‘gold-standard’ PD as follows: First, yearlong participation in a monthly content-focused seminar series and monthly collaboration with content specialists to develop the actual lesson plans. Second, as the module was implemented in the classroom, close interactions with the same science partner who shepherded the MFF PD in this study. These interactions involved in-person meetings several times a week, classroom observations and post-hoc oral and written reflections after each lesson, focused on the critical instructional components the module in particular the activities, discussions and questions. The outcomes of this training were then compared with outcomes of the MFF PD Program described below:

### Modeling for Fidelity (MFF)

The MFF PD program is structured in three parts (Table 1):

**Table 1:** Structure of the Modeling for Fidelity Professional Development Program.

Type of Support	Format	Goals
**Asynchronous**	**Educative materials available on line:**	
	Teacher Text	Provides content contextualized to lessons.
	Lesson Plans Narrative	Provides lesson structure and materials; Models Socratic Discussions.
	Student workbooks	Provides additional literacy opportunities; Addresses critical concepts and misconceptions; Extends problem-solving opportunities
**Synchronous virtual**	**Virtual interactions with science partner:**	
	Contextualized Content tutorials	Provides opportunity to review content and prepare for lessons
	Just-in-time-support	Provides opportunities to supplement and adjust teaching strategies; Provides opportunities to reflect on lessons
**Asynchronous**	**Living materials available on line:**	
	Teacher scrapbook	Provides heads' up of how to preserve critical instructional and structural components for fidelity of implementation
	Videos of model lessons	Provides insight into how to manage novel teaching strategies
	Discussion forum	Allows direct teacher-to-teacher communication

#### 1) The asynchronous educative texts and printable materials

These comprise a teacher content text, content contextualized lesson plans and student workbooks. The teacher text was written by the neuroscience content specialists at an early graduate school level and is targeted to teachers of high school biology, providing an up-to-date background in the underlying scientific information that parallels the curriculum. It also incorporates study guides designed to aid teacher learning as they progress through the material. Paired with the teacher text are content-contextualized lesson plans for each of the 27 lessons in the module. Each lesson follows the same basic structure – a ‘Do Now’ to introduce the topic and uncover misconceptions; an ‘Activity/Discussion’ that comprises the majority of the lesson and a ‘Wrap Up’ that contextualizes the lesson and reviews the learning objectives. Embedded in the lesson plans is a narrative that specifically models how the Socratic discussions might evolve in the classroom. Not only does this narrative contextualize the critical components of the learning goals, it also emphasizes key crosscutting concepts, core ideas and components of authentic scientific practice required by the NGSS [16], while supplying numerous examples that place the content in a life-relevant health-related context. Finally the module provides workbooks for the students. The workbooks also parallel the lessons and provide extended literacy opportunities with text-based questions, expanded explanations of key concepts and misconceptions and critical thinking questions. Teachers are advised to read the text, complete the study guides and be familiar with the lesson plans, narratives and student workbooks before entering the virtual part of the MFF program, in which they are paired with a science partner who works with them to facilitate implementation in the classroom.

#### 2) The synchronous virtual support component

These comprise prolonged mentoring interaction with a science content specialist through contextualized content tutorials, ‘just-in-time’ support and lesson reflections. Science partners offer support to piloting teachers as they prepare to implement the module in their classrooms and while they are teaching it. When preparing for lessons, teachers meet formally with their science partners over roughly a two-month period, which included the 6 weeks of curriculum implementation. The main focus of these tutorials is to ensure teachers are thoroughly prepared to teach the upcoming unit by reviewing the health science content, alerting teachers to common misconceptions, forecasting student questions, introducing lesson pedagogies and devising strategies to encourage active learning, tailored to each teacher's needs. One of the biggest challenges of implementing new curricula, especially GD, is making larger connections between individual lessons, units and even modules [16] [41]. The science partners are able to ensure that teachers appreciate these overarching connections. The individually tailored mini-seminars take place via virtual formats like gchat. On average, each unit requires one to three hours of tutorial time and can involve up to 10 participants. The flexibility of the virtual format means that mentoring can occur whenever is mutually convenient. Another synchronous support scaffold directed toward lesson preparation and implementation involves ‘just in time’ interactions between teachers and science partners to provide on-demand support with additional background, answering questions and adjusting teaching strategies. Just-in-time support is unscheduled and informal and takes place through text messaging emails and phone calls with the science partner readily available to answer last minute questions.

Real-time reflection is key to successful implementation [17], [42], [43] and thus is a key component of the synchronous support scaffold. Reflections are eventually compiled into a “scrapbook” of teacher feedback, used by the program to adjust lesson plans and inform other teachers (Table 2). This scrapbook is instrumental in archiving the experiences of the cohort of teachers with experience in the curriculum but whose geographic separation precludes interactions between them. The reflections also provide researchers with information on fidelity of implementation. The total time commitment for the synchronous component of MFF is approximately 32 hours (2 months ×4 hours/week) which includes tutorials, just-in-time-support and reflections. In contrast the time commitment for ‘gold standard PD′ was around 96 hours (48 hours content preparation and then lesson preparation at 6 hrs/week for 8 weeks which included 2–3 classroom observations, reflections and preparation time). Hence reducing the ‘dose’ of intervention significantly with MFF did not compromise outcomes.

**Table 2:** Example from teacher scrapbook for Unit 2.

Unit 2: What are the building blocks of our brains? Unit 2 is consistently ranked by students (and teachers) as the most challenging unit within the neuro module. I wouldn't argue with that ranking because Unit 2 includes perhaps the most complicated concept within the field of neuroscience – the action potential. The action potential is presented in Lesson 2.2, and up until that lesson, the students haven't really grappled with any concepts that are that far beyond everyday experiences and/or prior knowledge. So, take that as a warning – Lesson 2.2 is the end of the “honeymoon” phase with the content for this module. That lesson is certainly the most challenging, and things get easier again from there, but the concepts do continue to build on each other. This unit also dives down from talking about the brain as a whole (as we did in Unit 1), to focus on the structure and function of the cells of our nervous system – neurons and glia. So, before beginning this unit, you might want to help the students transition from talking about the brain as a whole to talking about the cells of the brain.	
Below are the comments/suggestions/feedback from other teachers about the specific lessons.	
**Lesson 2.1- What is the structure of a neuron?**	• This lesson tends to go really well with students who have not seen neurons and thus are unfamiliar with neuronal structure. It may seem a little elementary for those students who are already familiar with the main structures of a neuron and their functions. So, if you have a class that has already studied neurons, you may want to move through this material quicker to get started on laying the groundwork for the action potential. For example – you could start reviewing the concepts of diffusion and electrostatic pressure.
	• The activity within this lesson has students working in groups to create a variety of neuronal pathways. Basically, they're designing the different neurons/circuits that would complete different bodily functions for our friend Joe as he goes about his day. Students should be able to demonstrate that different parts of the neurons in their pathway have specialized structures to complete the necessary functions. You may choose to have student groups draw out their pathways, or alternatively, they could use clay to literally build the pathways.
**Lesson 2.2 –How do our axons transmit electrical signals?**	• This lesson has two different versions – a “differentiated” one and a non-differentiated one. The “differentiated” version contains many more details than the non-differentiated one. I recommend that for classrooms where students are still learning about the forces of diffusion and electrostatic pressure, and the impermeability of the cell membrane, that you use the non-differentiated (more basic) approach. But, there's nothing saying that you couldn't start there and build to the differentiated version. The workbook contains all the details included within the differentiated version, so if you're opting to use the more basic version with your students, you may want to warn them that their workbooks will give them many more details which they won't necessarily be responsible for (that's your choice in what you decide to test them on).
**Lesson 2. 3 –How fast do your neurons signal?**	• Students start the lesson by completing the ruler drop test to measure reaction time, and then from there calculate the speed of neuronal conduction. There are two versions of the Do Now worksheet – one in which students need to solve the formula d = 1/2at^2^ for t, and the other includes a table for the students to look up their reaction times based on distance the ruler dropped. Use the version that you think your students will handle best, and make sure students do the ruler drop test several times to be able to get their average reaction time.
	• Some teachers have noticed that this lesson can run short, whereas others have noticed it can run long. It all depends on how much time you allow for the do now, and how much discussion you get going around myelinated vs. un-myelinated, and the teenage brain.
**Lesson 2.4 – How do our neurons transport materials?**	• From this video, we're asking students to make observations about how the marked vesicles are moving. You'll probably have to clue them in with questions like – Are the vesicles always moving? What about what direction – always one speed or multiple? What about their speed – are they all going the same speed or different?
**Lesson 2.5 – What can go wrong?**	• We use a jigsaw and have the students break into groups to read different case studies of patients with disorders that are caused by neuronal dysfunctions. I've heard from many teachers that the cases are approachable to their students, and that the students tend to like this lesson best of all of those within Unit 2, so they end on a high note.

#### 3) The asynchronous online educative component

The final support scaffold comprises materials available to teachers online that support lesson planning and implementation, each meeting a unique goal that further facilitates MFF. First, there are videos of teachers in their classrooms enacting lessons that use unique pedagogical approaches or where the content is particularly challenging. These videos allow novice teachers to sample various teaching strategies in the context of a “live” classroom. Second there is a discussion forum that creates an online community where teachers can communicate about curriculum implementation. Finally there is a news blog maintained by the program that features news stories that relate to the content. The news blog allows both teachers and students see the real-life implications of the information presented in the classroom.

## Results

The MFF PD program was designed to support a comprehensive biomedical and health sciences high school curriculum that focuses on cutting edge topics in health and disease and is structured to foster student capacities in both health science and health literacy, topics that high school teachers are rarely exposed to. Given this challenge, MFF had to: a) Incorporate current best practices in professional development; b) Build sufficient content knowledge so the teacher could enable their students to access the material, draw connections between cross-cutting concepts and to evaluate health claims and risk perception critical to fostering health literacy capacities; c) Contextualize the content knowledge to a variety of pedagogical strategies that would make the material accessible to a wide variety of learners; d) Empower teachers to adapt the pedagogy to their classrooms while preserving critical concepts; e) Allow access that was not constrained by the geographic proximity of teachers relative to the program site in Boston Massachusetts. To address (a) and (b) the program focused on establishing a prolonged mentoring relationship between the teacher and a biomedical science content specialist ‘science partner’. To address (c) the program provided the teacher with a comprehensive set of educative materials that had been developed by a collaborative learning community of scientist content experts and high school teachers. To address (d) and (e) the program utilized a hybrid online/virtual format in which elements of personal interaction were established on a background of downloadable, web-accessible materials.

### Teacher outcomes

Teacher responses to the MFF format were assessed with an anonymous survey. The survey with sample responses is reported in Table D in S1 File. A complete list of the survey responses is provided with the Supporting Materials (S1 File). A major goal of MFF is, as one teacher phrased it, not to leave the teachers ‘alone’ in the classroom even though they are geographically removed from their science partner. The face-to-face aspect of the virtual mentoring interactions provided by the gchat format meant teachers and mentors were able to establish the personal connections and trust that purely online interactions are not likely to provide. In fact, only one teacher weakly preferred in-person interactions (Table D in S1 File):

‘*Skype and Google Chat worked well, but I would have loved to be able to meet with someone in person, even if it was prior to or after implementation of the curriculum*’.

**Figure 1:**
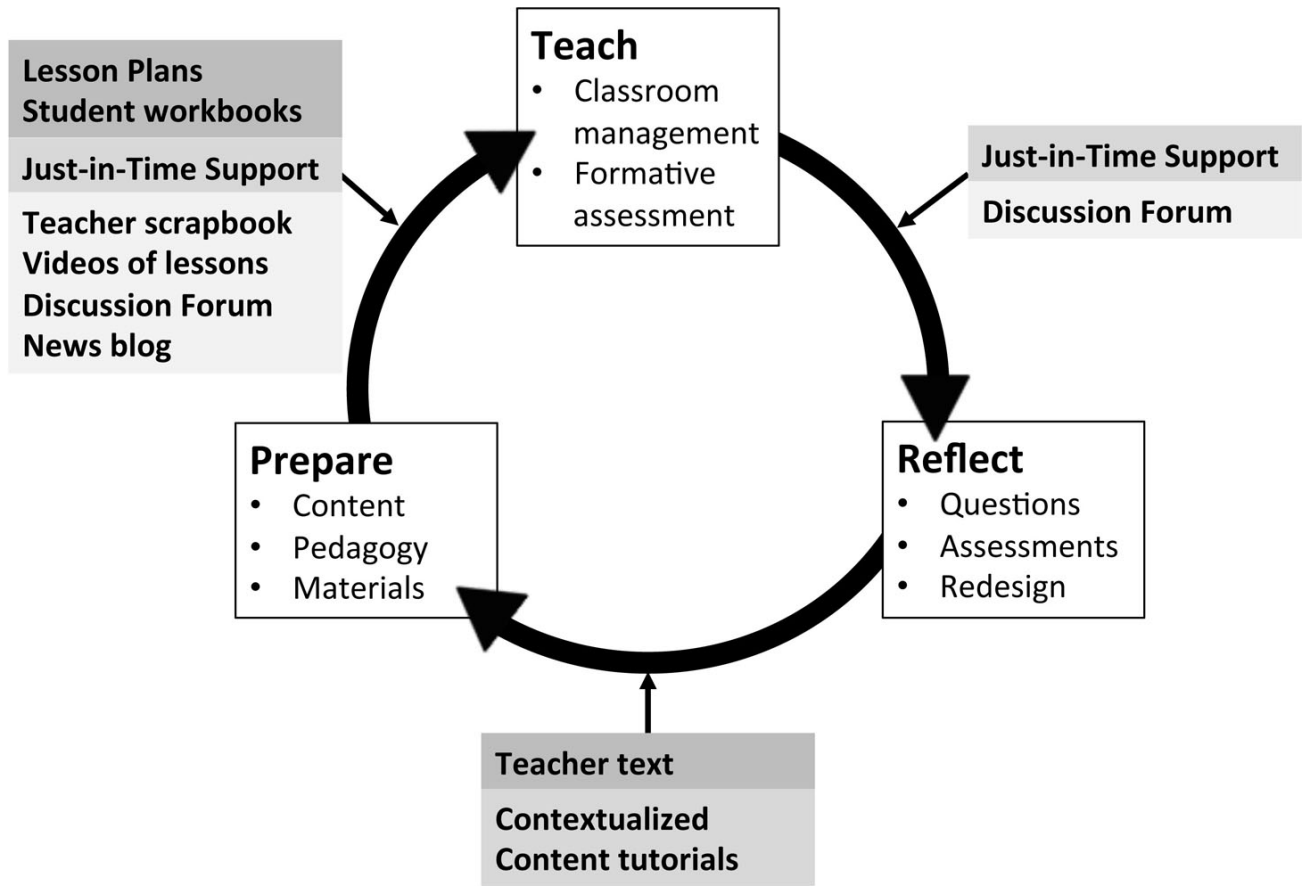
How Modeling for Fidelity is used. The process of curriculum implementation has three stages - ‘prepare’ ‘teach and ‘reflect’ that occur iteratively on a lesson, unit and module basis. Modeling for Fidelity support scaffolds are integrated into each stage as follows: The asynchronous online lesson plans and student workbooks are primarily used during preparation for teaching, while the teacher text is used during reflection after teaching to address student questions. The synchronous virtual mentorship interactions during contextualized content tutorials are used during initial preparation, while just-in-time support is used to field questions while the teacher is involved in teaching in the classroom. Finally the asynchronous live materials found in the teacher scrapbook, videos, discussion forum and news blog are all ways to connect with prior teachers' experiences.

Curriculum implementation involves an ongoing cycle of lesson preparation, presentation and reflection (Fig. 1). Teachers first used the contextualized content tutorials with their science partners to review the Unit level material in the teacher text. The structure of this review emerged from the yearlong immersion in scientific content the ‘gold standard PD′ teacher experienced during the module design process. After this overview teachers and science partners then turned to the lesson narratives and student workbooks to familiarize themselves with the pedagogical approaches and physical materials used in each lesson as well as the videos modeling teaching strategies. Teachers also consulted online discussion forums for other teachers' suggestions. MFF participants judged the contextualized content tutorials as one of the most useful and valuable parts of our PD approach with respect to fidelity of implementation (Table D in S1 File):

‘*I believe that as a teacher, I have the expertise and ability to implement my curriculum with my students, however I sometimes feel that my content knowledge is lacking. The support that I find that I need as a teacher is support that deepens my understanding and extension of content. Rather that (sic) constantly working to try to re-learn or re-teach all the material to myself, it was incredibly helpful to have a mentor who I could rely on and interact with regularly. I didn't feel so "alone" in my classroom*’.

‘*Having a "live" person to walk through each unit before I introduced it to my class was like having my own mini college throw back seminar. Or better than that, it is like a (finally) useful in-service where teachers are introduced something that seems theoretical AND also given the practical application for the classroom. Wow! What a concept*’.

As the module began to be taught teachers could get additional help via informal just-in-time support from their science partner. The just-in-time support scaffold emerged in response to the productivity of the informal interactions between teacher and science partner during ‘gold standard’ PD, so as the teachers began to teach the module in the classrooms they were encouraged utilize this on-demand support and be in touch via email, phone and text messaging as-needed. These serendipitous communications were often useful for instructional components such as classroom discussions. For example, during one sheep brain dissection lab, the brain displayed an abnormal growth near the cerebellum. The teacher messaged a picture to the science partner who was able to confirm the teacher's predictions that it was most likely a tumor, provoking further in-depth discussion among the class. It maybe worth noting that the willingness of the teacher to seek a second opinion from the science partner while in the midst of a lesson illustrates teacher-mentor trust. Not surprisingly teachers also found this just-in-time access an expert critical to fidelity of implementation and valued it highly (Table D in S1 File):

‘*I think that the most useful aspect of the support was the regular access to an expert. If I encountered questions or extensions in my preparations or in my teaching, I always had an expert ready and eager to help*.’

We had hoped that the teachers would utilize the support scaffolds provided in MFF on a regular basis, and this was indeed the case. The cycle of ‘prepare, teach and reflect’ continued on both unit and lesson levels as the curriculum progressed through implementation. Just-in-time support after each lesson helped teachers with student questions they could not answer. The information collected from the lesson questionnaires and information about how and why teachers had adapted lessons was aggregated into the online scrapbook (see Table 2).

### Student outcomes

One critical aspect of the Fidelity of Implementation protocols employed during ‘gold standard’ PD that could not be replicated following MFF was classroom observation. We circumvented this inability with an indirect, yet highly relevant measure of implementation, namely objective assessment of student outcomes. We employed the same measures of student achievement used to initially determine efficacy of the ID (Infectious Disease) module, namely gains in conceptual knowledge inventory and problem solving skills related to health science literacy, attitudes towards the material, and finally self-efficacy in learning, a critical metric for developing health literacy capacities [14].

**Figure 2:**
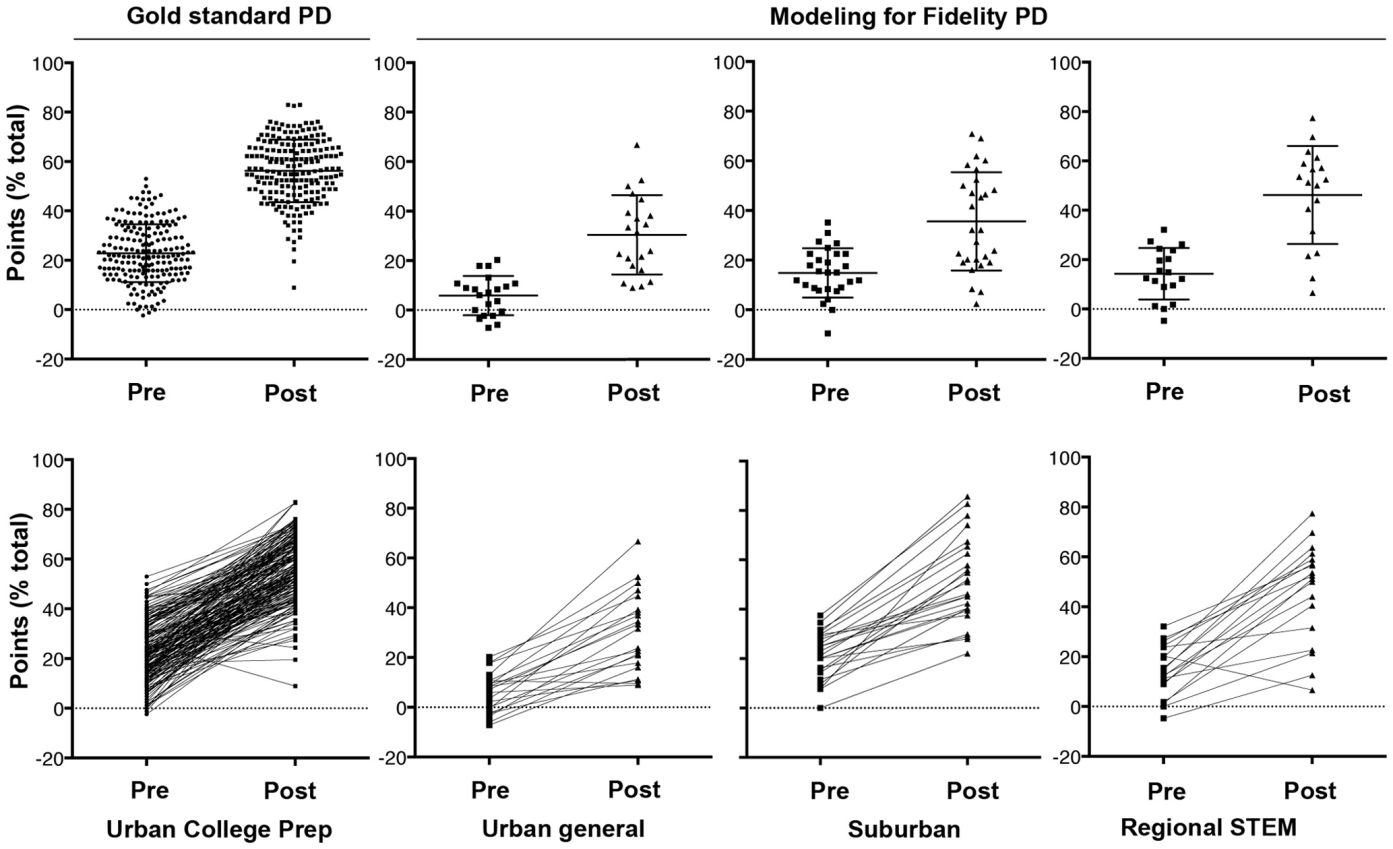
Grouped and paired comparison of individual student gains in conceptual knowledge inventory and problem solving skills relevant to neurological disorders. Top panel: Student scores (%) following the ND conceptual content knowledge inventory and problem-solving skills pre- and post-tests plotted as mean (±) SD by school. The description of each school appears below. The panel on the left represents a school whose teacher experienced the intensive in-person professional development program we term ‘gold standard’. The three panels on the right indicate schools whose teachers had experienced the ‘Modeling for Fidelity’ professional development program. See Table 3 for numerical data. In each case the pre-post difference was significant p<0.0001, however there was no difference between any of the schools (ANOVA). Bottom panel: Student scores (%) following the ND conceptual content knowledge inventory and problem-solving skills pre- and post-tests plotted as mean (±) SD by school, disaggregated to show individual students' gains.

*Conceptual knowledge inventory and problem-solving skills:* Fig. 2 shows the individual paired pre-and post-test conceptual knowledge inventory scores. Table 3 provides the numerical data for the groups by school. Even though average pre- and post-test scores were highly variable among schools (p<0.001, ANOVA) the pre- to post-change within each individual school was highly significant (p<0.0001, paired t test). The fold post-pre change and effect size were both largest in the ‘gold standard’ school, but these values were not significantly different from the MFF schools. The schools represent a range of demographics. To determine how this impacted outcomes, student data was replotted individually by school, illustrating that the few (2/273) students who failed to demonstrate gains were clustered in the urban general high school (Fig. 2).

**Table 3:** Conceptual knowledge inventory and problem solving skills.

School Setting	Pre-test Mean (SD)	Post-test Mean (SD)	Paired Fold Change (SD)	Cohen' ‘d’
Comparison Teacher (Urban Exam)	19.14 (7.46)	65.28 (13.00)*	3.90 (1.88)	4.35
MFF Teacher 1 (Urban General)	11.27 (7.62)	37.37 (13.93)*	3.71 (1.89)	2.32
MFF Teacher 2 (Suburban General)	21.43 (9.56)	51.32 (17.64)*	2.80 (1.87)	2.11
MFF Teacher 3 (Regional STEM)	18.93 (9.57)	60.14 (12.00)*	3.52 (2.00)	3.80
Total (All schools)	18.63 (8.35)	60.03 (16.35)*	3.70 (1.91)	3.19

**Pre-post gains in concept inventory and problem solving skills relevant to evaluating health claims in neurological disorders.** Student gains in ND concept inventory were evaluated from 10 multiple-choice and 2 short answer questions. Problem solving skills were evaluated from 5 case study questions. Pre-post differences were measured by paired t test and were significant (*p<0.0001). Effect size measured by Cohen's ‘d’ is very high (N = 175 total students from 4 schools).

To illustrate pre-posttest changes in student comprehension, we provide short answers from a student who scored at the average of the cohort on the pre- and post-test. The responses are to a question in the clinical case study: ‘*One of the reasons Sober in Somerville thinks he is not an alcoholic is because he believes he could stop drinking at any time. Explain which areas of his brain control his compulsions to drink*.’

*Pretest response: ‘The back part of his brain’.*

*Posttest response: ‘The parts of the brain's reward pathway: VTA (ventral tegmental area), NAc (nucleus accumbens) and PFC (prefrontal cortex). When he is drinking alcohol, there is a release of dopamine from the VTA to NAc’.*

Together the data demonstrate that the core ND conceptual knowledge inventory and claims evaluation was accessible to a diverse student demographic, including English language learners, regardless of whether teachers received ‘gold standard’ or MFF PD. Moreover the type of PD experienced by the teachers does not affect student achievement.

#### Attitude and self-efficacy

Our underlying rationale for developing the GD curriculum was that students will engage with material they find inherently valuable, thereby improving their academic performance. To begin to assess how students perceive ND we measured student attitudes towards the material after the course was completed with an online anonymous survey (Table D in S1 File).

**Figure 3:**
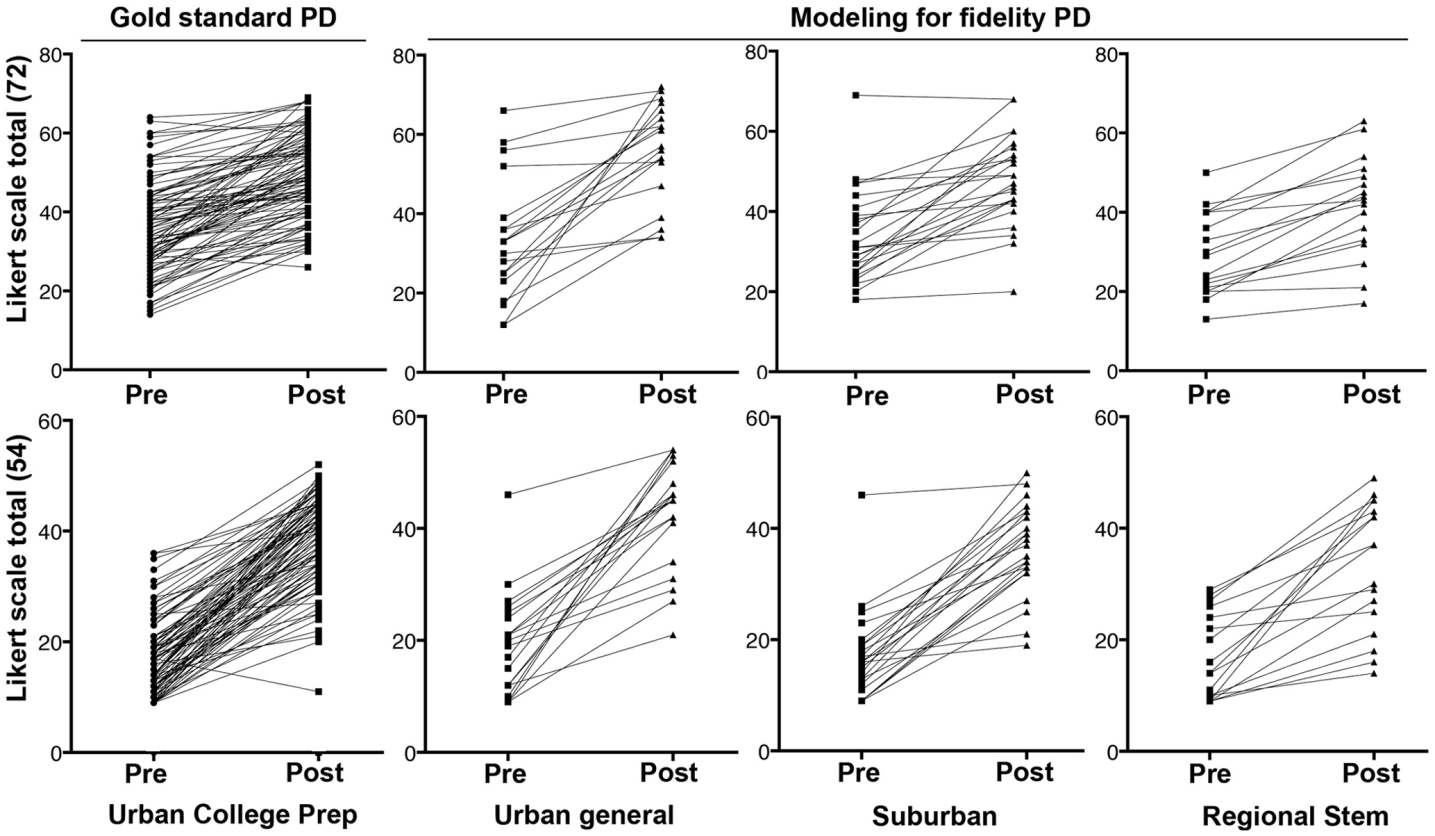
Grouped and paired comparison of individual student gains in attitude and self efficacy toward learning about neurological disorders. Top panel: Student self-reported changes in attitude towards studying neurological disorders following a retrospective-pre/post Likert scale survey (54 points total) disaggregated to show individual students' gains. The description of each school appears below. The panel on the left represents a school whose teacher experienced the intensive in-person professional development program we term ‘gold standard’. The three panels on the right indicate schools whose teachers had experienced the ‘Modeling for Fidelity’ professional development program. See Table 4 for numerical data. Pre-post differences were measured by paired t test and were significant (p<0.0001), however there was no difference between any of the schools (ANOVA). Bottom panel: Student self-reported changes self-efficacy towards studying neurological disorders following a retrospective-pre/post Likert scale survey (54 points total) disaggregated to show individual students' gains. The description of each school appears below. The panel on the left represents a school whose teacher experienced the intensive in-person professional development program we term ‘gold standard’. The three panels on the right indicate schools whose teachers had experienced the ‘Modeling for Fidelity' professional development program. See Table 4 for numerical data. Pre-post differences were measured by paired t test and were significant (p<0.0001), however there was no difference between any of the schools (ANOVA)

Fig. 3 shows the individual paired pre-and post-test conceptual knowledge inventory scores. Table 4 provides the numerical data for the groups by school. Data showing changes in attitudes following participation were normally distributed (Kolmogorov-Smirnov). Average retrospective-pre and post-test scores and fold change were less variable between schools than conceptual knowledge inventory data, but were not significantly different. Again the fold change within each school was highly significant (p<0.0001, paired t test). Likewise the effect size for each school was large measured by Cohen's d (from 1.03 to 1.53) and was not significantly different regardless of the type of PD the teacher experienced. Hence the PD experienced by the teachers does not affect student attitude to the material.

**Table 4:** Attitudes toward content.

School Setting	Pre-test Mean (SD)	Post-test Mean (SD)	Paired Fold Change (SD)	Cohen's ‘d’
Comparison Teacher (Urban Exam)	35.86 (11.40)	49.14 (10.36)*	1.45 (0.38)	1.22
MFF Teacher 1 (Urban General)	34.40 (15.85)	56.05 (12.24)*	2.01 (1.21)	1.53
MFF Teacher 2 (Suburban General)	33.67 (11.62)	47.92 (11.24)*	1.51 (0.42)	1.25
MFF Teacher 3 (Regional STEM)	29.47 (10.45)	41.47 (12.80)*	1.45 (0.30)	1.03

**Retrospective pre-posttest self-reported gains in attitude relevant to the study of neurological disorders.** Students were evaluated for their self-reported changes in attitude with a retrospective pre-post test analysis. The survey had a total of 54 possible points. Pre-post differences were measured by paired t test and were significant (*p<0.0001). The effect size was measured via Cohen's ‘d’ is very high. (N = 147 total students from 4 schools).

An additional measure critically related to the health literacy goals of the curriculum is student self-efficacy, which is students' perception that they have the capacity to learn about a topic. The fold change between schools was particularly consistent (2.22–2.68) and highly significant (p<0.0001, paired t test) within each school, and in this case, not the largest in the ‘gold standard PD’ school. Effect size for each school measured by Cohen's d was large (Table 5). Plotting student gains in self-efficacy individually by school revealed that the 3/147 students who failed to demonstrate gains were distributed among the ‘gold standard’ school (1) and the suburban general school (2). These data indicate that participating in the curriculum significantly improves students’ perceptions of their ability to learn about ND a critical element in initiating the life-long learning required for health literacy, and these gains occurred irrespective of the type of PD their teacher has experienced.

**Table 5:** Self-efficacy.

School Setting	Pre-test Mean (SD)	Post-test Mean (SD)	Paired Fold Change (SD)	Cohen's ‘d’
Comparison Teacher (Urban Exam)	17.29 (7.00)	37.80 (8.71)**	2.44 (0.86)	2.60
MFF Teacher 1 (Urban General)	19.25 (9.13)	42.75 (9.66)**	2.68 (1.41)	2.50
MFF Teacher 2 (Suburban General)	16.79 (7.91)	36.08 (7.99)**	2.42 (0.84)	2.43
MFF Teacher 3 (Regional STEM)	16.88 (7.66)	33.29 (11.59)**	2.22 (1.05)	1.70

**Retrospective pre-post test self-reported gains in self-efficacy relevant to the study of neurological disorders.** Students were evaluated for their self-reported changes in self-efficacy with a retrospective pre-post test analysis. The survey had a total of 54 possible points. Pre-post differences were measured by paired t test and were significant (*p<0.0001). The effect size was measured via Cohen's ‘d’ is very high. (N = 147 total students from 4 schools).

### Within-teacher comparison of PD programs

One of the MFF teachers had also experienced ‘gold-standard’ PD with a different scientist partner in preparation for teaching the Infectious Disease (ID) module of the GD curriculum to the same group of students from the urban general high school. Comparing their outcomes after participating in both modules (Table 6) shows significant gains in all of the measures analyzed, regardless of the type of PD their teacher experienced. The only measure significantly different between ND and ID was self-efficacy (Cohen's d 3.03 and 1.53 respectively). Whether this larger gain following ND may reflect a dose response effect since students experienced the ID module first deserves further investigation.

**Table 6:** Within-teacher comparison.

Urban general school	Pre-test Mean (SD)	Post-test Mean (SD)	Paired Fold Change (SD)	Cohen's ‘d’
Conceptual knowledge ‘Gold standard’ support (ID)	5.84 (7.93)	30.36 (16.06)		1.94
Conceptual knowledge MFF (ND)	11.27 (7.62)	37.37 (13.93)	3.71 (1.89)	2.32
Self efficacy ‘Gold standard’ support (ID)	18.83 (7.91)	41.48 (8.16)		3.03
Self efficacy MFF (ND)	34.4 (15.85)	56.05 (12.24)	2.01 (1.21)	1.53

**Within teacher comparison between ‘gold standard’ and ‘modeling for fidelity’ professional development programs.** The urban general high school teacher taught two modules from the GD curriculum to the same set of students. The first module, ID, was prepared with ‘gold standard’ PD, while the second module was prepared with MFF (the modules had different science partners). The effect size measured via Cohen's ‘d’ was very high in all cases and students made highly significant gains (p<0.0001, paired t test) in both measures regardless of the mode of PD their teacher had experienced.

## Discussion

Biomedical content specialists, typically associated with research medical institutions, act as gatekeepers for the kind of cutting edge health science that mirrors student real-world experience [14]. There is little professional incentive for them to engage with K-12 education, and as a consequence, this type of health science is largely inaccessible both to students and their teachers. Our previous project ‘A Collaborative Approach to Real World Science in the Classroom’ used ‘best practices’ for collaborative curriculum design to create a health science curriculum focused on ‘Great Diseases’ of global significance. The experience taught us that making this kind of material accessible requires intensive and prolonged interactions between biomedical content specialists and teachers [14]. This observation led to the development of a program of long-term teacher mentoring by science partners in the context of curricula implementation. The purpose of this study was to determine whether key elements of the mentoring relationship could be migrated to a scalable virtual environment in which the parties could interact without geographic limits.

MFF was assessed with respect to how well it promoted fidelity of implementation of the ND module in the classroom directly, via self-reported teacher performance. Classroom observations, commonly a key component of fidelity of implementation protocols are precluded by MFF′s distance learning format [44]–[47]. Fidelity of implementation was also measured indirectly via student performance. Student outcomes are arguably the critical benchmark of successful implementation, but are rarely reported in PD studies [48]–[51] possibly because they are difficult to interpret when common practices are used in different classrooms with different curricula. This study, by assessing outcomes in the context of a common curriculum, was able to circumvent that problem.

Student outcomes were similar, regardless of whether the teacher received in-person training or interacted with their science partner virtually. Exposure to the curriculum significantly increased students' conceptual knowledge and problem solving skills required to form a framework for understanding current and future issues in ND. Conceptual knowledge is a recognized element of health literacy, but understanding when conceptual knowledge about disease is most effectively acquired is underexplored [52]. This data showed that the conceptual knowledge and problem solving skills considered by the ND experts as essential for establishing a framework for ND health literacy, was accessible to students at the high school level. Moreover students understood the interactions between the core ideas and could use them to solve problems similar to those that they may encounter in a clinical healthcare setting, skills also demanded by current science standards [16], [40]. Additionally, the curriculum also increased student self-efficacy and attitude. Medical advances are rapidly evolving, and adequate health literacy is dependent on ongoing independent learning. Belief in one's capacity to learn about a topic (self-efficacy) is highly correlated with actual capacity to learn [53]–[55] and is a critical element of ongoing commitment to independent learning [53]–[57]. Our results showed that prior to taking the course, students assessed their capacity to learn the ND material as low, consistent with the lack of any prior health-science literacy related accomplishments that would reinforce self-efficacy of learning [53]. However, after participating in the curriculum, students' perceptions of their capacity to learn about ND increased significantly, demonstrating that the high school biology classroom is an underexploited setting in which to improve student attitudes towards their capacity to learn skills important for managing their health in a dynamic healthcare landscape [53]–[57].

Although high quality professional development that is content-focused is possible in a purely online format [29], [50], [58] ‘best practices’ of online PD rarely incorporate active learning techniques or a prolonged mentor relationship with a content expert or measure student outcomes 17,26,32]. MFF incorporates opportunities for sustained virtual interactions into more traditional online educative materials, producing equivalent results to intensive ‘gold standard’ PD and improving teacher practice. As one of the piloting teachers put it when asked how participating in MFF had impacted practice outside of the ND module:

‘*I break up my lessons. I search for ways to have the kids do more. I've incorporated watching You Tube videos on things such as split brain and brain anatomy as homework (which they LOVE). I've made up my own case studies similar to the "what could go wrong" lab. In sum, the partnership between a professional in the classroom and a professional in the field who really gets teaching in absolutely invaluable. Absolutely. This was an amazing discovery for me and enhanced both my class and my teaching practices overall*.’

The teachers included in this case study have significant differences in experience and teach a wide range of student demographics and abilities, yet most of their students demonstrated equivalent and significant improvements in conceptual knowledge and problem solving skills, attitudes and self-efficacy. Hence the MFF approach allows different teachers in diverse high schools to elicit strong student performance, whether they serve English language learners, are college preparatory, STEM focused or serve a variety of students in a suburban setting. It will be important to try to gather more information reflecting the interaction between teacher heterogeneity and PD with student outcomes, but this kind of analysis will require a larger teacher cohort.

Our approach to curriculum implementation differs from others that focus on establishing a rigid fidelity of implementation in the classroom [44]–[47]. The curriculum was designed in partnership with teachers with the philosophy that if teachers displayed mastery in content and an in-depth understanding of the critical components of the learning objectives, they should feel empowered to adapt lessons for their individual classrooms and to share the rationale for the adaptation with us [59]. Overall this strategy helped to establish trust between teachers and their science partners and led to a more accurate reporting of implementation, which may be lost when teachers feel pressure to deliver lessons in a prescribed way. Teacher adaptation of the materials fell into two general categories: tailoring the context of a specific concept to the specific interests of their students, or adapting the teaching method to best fit the learning needs of their classrooms. Interacting with the scientist partner meant that any changes made retained scientific accuracy and maintained the key learning objectives, as the consistency of student outcomes revealed.

We also want wanted to probe whether MFF encouraged teachers to use an active form of inquiry-based learning in their classes. Teachers were guided to this approach through the educative materials and content trainings, particularly the lesson narratives and videos, which modeled active inquiry based on extensive Socratic discussion. The fact that teachers largely followed these instructional approaches even though they were encouraged to adapt the lessons suggests that the program successfully evoked these practices. Notably the teachers in this study have utilized the module in successive semesters or academic years, continuing their interactions with the science partner, further confirming that they deem these elements of practice valuable. Interestingly there have also been significant increases in the numbers of students taking this elective course since the GD curriculum was introduced in the urban exam and general schools, confirming both the relevance of health science to teenagers and the facility of the teachers with the curriculum. Nonetheless an inherent limitation of MFF, like other PD programs is that the participants self-selected to use the materials and participate in the training program after assessing the curricular materials online. Whether significant numbers of teachers who are not comfortable with an active inquiry approach may be dissuaded from participating even before building the key relationship with a science partner is not clear.

### Conclusions and Future Directions

The data presented here make three important contributions to research about Professional Development: First PD programs that incorporate virtual interactions between a teacher and a science partner by incorporating layers of asynchronous and synchronous interactions over an extended period of time can produce outcomes like intensive ‘gold standard’ PD. Second, that teachers embrace this method of PD and value it particularly for its focus on rigorous contextualized content and on-demand support, Third, that this PD approach makes novel content accessible to diverse teachers who can in turn transfer the content into their classrooms to elicit significant gains in their students. The data also indicate that teaching about health in high school is an excellent vehicle to address poor student engagement in science. To be engaged in learning, students must perceive the content they are being asked to learn as valuable 5,7,8,10]. Given the broad cultural relevance of health, health sciences, like ND, provide an opportunity to leverage topics inherently valued by diverse students to elicit robust student engagement in science [14] as well as to open a new avenue to foster HL education. Our future goal is to further streamline MFF to increase scalability, strengthening the asynchronous training materials and building a teacher network to foster teacher-teacher interactions in high school biology classrooms across the country.

## Supporting Information

S1 File **Supporting Figures and Tables providing extended datasets.**
(PDF)

## References

[1] OECD (2014) PISA 2012 Results: Creative problem solving: Students' skills in tackling real-life problems (Volume V). Available: http://dx.doi.org/10.1787/9789264208070-en Accessed April 22, 2014.

[2] Provasnik S, Kastberg D, Ferraro D, Lemanski N, Roey S, et al (2012) Highlights from TIMSS 2011: Mathematics and Science Achievement of US Fourth-and Eighth-Grade Students in an International Context. NCES 2013-009. National Center for Education Statistics Available: http://ncesedgov/pubs2013/2013009_1pdf Accessed April 22, 2014.

[3] National Center for Education Statistics (2012) The Nation's Report Card: Science 2011 (NCES 2012-465). Available: http://nces.ed.gov/nationsreportcard/pdf/main2011/2012465.pdf. Accessed April 22, 2014.

[4] Bao L, Cai T, Koenig K, Fang K, Han J, et al (2009) Learning and scientific reasoning. Science 323:586–587. 19179514 10.1126/science.1167740

[5] Deci EL, Vallerand RJ, Pelletier LG, Ryan RM (1991) Motivation and education: The self-determination perspective. Educational psychologist 26:325–346.

[6] Vansteenkiste M, Lens W, Deci EL (2006) Intrinsic versus extrinsic goal contents in self-determination theory: Another look at the quality of academic motivation. Educational psychologist 41:19–31.

[7] Hidi S (1990) Interest and its contribution as a mental resource for learning. Review of Educational Research 60:549–571.

[8] Hagay G, Baram-Tsabari A, Ametller J, Cakmakci G, Lopes B, et al (2013) The generalizability of students' interests in biology across gender, country and religion. Research in Science Education 43:895–919.

[9] Renninger K, Hidi SE, Krapp AE (1992) The role of interest in learning and development: Lawrence Erlbaum Associates, Inc.

[10] Tobias S (1994) Interest, prior knowledge, and learning. Review of Educational Research 64:37–54.

[11] Sandoval J (1995) Teaching in subject matter areas: Science. Annual Review of Psychology 46:355–374.

[12] Malone TW, Lepper MR (1987) Making learning fun: A taxonomy of intrinsic motivations for learning. Aptitude, learning, and instruction 3:223–253.

[13] Brooks JG (1999) In search of understanding: The case for constructivist classrooms: ASCD.

[14] Jacque B, Malanson K, Bateman K, Akeson B, Cail A, et al (2013) The Great Diseases Project: a partnership between Tufts Medical School and the Boston public schools. Acad Med 88:620–625. 23524931 10.1097/ACM.0b013e31828b50fb PMC3767121

[15] Cohen AK, Syme SL (2013) Education: a missed opportunity for public health intervention. American journal of public health 103:997–1001. 23597373 10.2105/AJPH.2012.300993 PMC3698749

[16] States NL (2013) Next Generation Science Standards: For States, By States Achieve, Inc. on behalf of the twenty-six states and partners that collaborated on the NGSS.

[17] Garet MS, Porter AC, Desimone L, Birman BF, Yoon KS (2001) What makes professional development effective? Results from a national sample of teachers. American Educational Research Journal 38:915–945.

[18] Shulman LS (1987) Knowledge and teaching: Foundations of the new reform. Harvard educational review 57:1–23.

[19] Shulman LS (1994) Those who understand: Knowledge growth in teaching. Teaching and learning in the secondary school: 125–133.

[20] Ball DL, Thames MH, Phelps G (2008) Content knowledge for teaching what makes it special? Journal of Teacher Education 59:389–407.

[21] Alexander D, Heaviside S, Farris E, Burns S (1999) Status of education reform in public elementary and secondary schools: Teachers' perspectives: US Department of Education, Office of Educational Research and Improvement. Available: http://nces.ed.gov/pubs99/1999045.pdf. Accessed April 22, 2014.

[22] Darling-Hammond L, Wei RC, Andree A, Richardson N, Orphanos S (2009) Professional learning in the learning profession. Washington, DC: National Staff Development Council Available: http://learningforwardorg/docs/pdf/nsdcstudy2009pdf Accessed April 22, 2014.

[23] Wei RC, Darling-Hammond L, Adamson F (2010) Professional development in the United States: Trends and challenges. Dallas, TX: National Staff Development Council Available: http://learningforwardorg/docs/pdf/nsdcstudytechnicalreport2010pdf?sfvrsn=0 Accessed April 22, 2014.

[24] Darling-Hammond L (1999) Teacher quality and student achievement: A review of state policy evidence: Center for the Study of Teaching and Policy, University of Washington Seattle, WA.

[25] Ball D, Cohen D (1999) Toward a Practice-Based Theory of Professional Education. Teaching as the Learning Profession San Francisco: Jossey-Bass.

[26] Penuel WR, Fishman BJ, Yamaguchi R, Gallagher LP (2007) What makes professional development effective? Strategies that foster curriculum implementation. American Educational Research Journal 44:921–958.

[27] Lieberman A (1995) Practices that support teacher development: Transforming conceptions of professional learning. Innovating and Evaluating Science Education: NSF Evaluation Forums 1992–94:67.

[28] Dede C, Ketelhut DJ, Whitehouse P, Breit L, McCloskey EM (2009) A research agenda for online teacher professional development. Journal of Teacher Education 60:8–19.

[29] Lock JV (2006) A new image: Online communities to facilitate teacher professional development. Journal of Technology and Teacher Education 14:663–678.

[30] King KP (2002) Identifying success in online teacher education and professional development. The Internet and Higher Education 5:231–246.

[31] Beyer CJ, Delgado C, Davis EA, Krajcik J (2009) Investigating teacher learning supports in high school biology curriculuar programs to inform the design of educative curriculum materials. Journal of Research in Science Teaching 46:977–998.

[32] Loucks-Horsley S, Hewson P, Love N, Stiles K (1998) Designing professional development for teachers of mathematics and science. Thousand Oaks, CA: Corwin Press.

[33] CEMSE (2014) Outlier Research and Evaluation. Available: http://outlier.uchicago.edu/. Accessed April 22, 2014.

[34] Pratt CC, McGuigan WM, Katzev AR (2000) Measuring program outcomes: Using retrospective pretest methodology. American Journal of Evaluation 21:341–349.

[35] Bhanji F, Gottesman R, de Grave W, Steinert Y, Winer LR (2012) The Retrospective Pre–Post: A Practical Method to Evaluate Learning from an Educational Program. Academic Emergency Medicine 19:189–194. 22320369 10.1111/j.1553-2712.2011.01270.x

[36] Skeff KM, Stratos GA, Bergen MR (1992) Evaluation of a Medical Faculty Development Program A Comparison of Traditional Pre/Post and Retrospective Pre/Post Self-Assessment Ratings. Evaluation & the Health Professions 15:350–366.

[37] Moore D, Tananis CA (2009) Measuring change in a short-term educational program using a retrospective pretest design. American Journal of Evaluation 30:189–202.

[38] Cohen S (1988) Psychosocial models of the role of social support in the etiology of physical disease. Health psychology 7:269. 3289916 10.1037//0278-6133.7.3.269

[39] Tanner K, Allen D (2004) Approaches to biology teaching and learning: learning styles and the problem of instructional selection—engaging all students in science courses. Cell Biology Education 3:197–201. 15592590 10.1187/cbe.04-07-0050 PMC533116

[40] National Research Council (2012) A Framework for K-12 Science Education: Practices, Crosscutting Concepts, and Core Ideas; Quinn H, Schweingruber H, Keller T, editors: The National Academies Press. Available: http://www.nap.edu/catalog.php?record_id=13165. Accessed April 22, 2014.

[41] Ball DL, Cohen DK (1996) Reform by the book: What is: Or might be: The role of curriculum materials in teacher learning and instructional reform? Educational researcher: 6–14.

[42] Darling-Hammond L, McLaughlin MW (1995) Policies that support professional development in an era of reform. Phi Delta Kappan 76:597–604.

[43] Schneider RM, Plasman K (2011) Science Teacher Learning Progressions A Review of Science Teachers' Pedagogical Content Knowledge Development. Review of Educational Research 81:530–565.

[44] Dusenbury L, Brannigan R, Falco M, Hansen WB (2003) A review of research on fidelity of implementation: implications for drug abuse prevention in school settings. Health Education Research 18:237–256. 12729182 10.1093/her/18.2.237

[45] O′Donnell CL (2008) Defining, conceptualizing, and measuring fidelity of implementation and its relationship to outcomes in K–12 curriculum intervention research. Review of Educational Research 78:33–84.

[46] Carroll C, Patterson M, Wood S, Booth A, Rick J, et al (2007) A conceptual framework for implementation fidelity. Implementation Science 2:1–9. 18053122 10.1186/1748-5908-2-40 PMC2213686

[47] Mowbray CT, Holter MC, Teague GB, Bybee D (2003) Fidelity criteria: Development, measurement, and validation. American Journal of Evaluation 24:315–340.

[48] Desimone LM (2009) Improving impact studies of teachers' professional development: Toward better conceptualizations and measures. Educational researcher 38:181–199.

[49] Fishman BJ, Marx RW, Best S, Tal RT (2003) Linking teacher and student learning to improve professional development in systemic reform. Teaching and teacher education 19:643–658.

[50] Fishman B, Konstantopoulos S, Kubitskey BW, Vath R, Park G, et al (2013) Comparing the Impact of Online and Face-to-Face Professional Development in the Context of Curriculum Implementation. Journal of Teacher Education 64:426–438.

[51] Borko H (2004) Professional development and teacher learning: Mapping the terrain. Educational researcher 33:3–15.

[52] Nielsen-Bohlman L, Panzer AM, Kindig DA (2004) Health literacy: a prescription to end confusion: National Academies Press. 25009856

[53] Britner SL, Pajares F (2006) Sources of science self-efficacy beliefs of middle school students. Journal of Research in Science Teaching 43:485–499.

[54] Basu A, Dutta MJ (2008) The relationship between health information seeking and community participation: The roles of health information orientation and efficacy. Health communication 23:70–79. 18443994 10.1080/10410230701807121

[55] Austin EW, Pinkleton BE, Austin BW, Van de Vord R (2012) The Relationships of Information Efficacy and Media Literacy Skills to Knowledge and Self-efficacy for Health-Related Decision Making. Journal of American College Health 60:548–554. 23157196 10.1080/07448481.2012.726302

[56] Zeldin AL, Britner SL, Pajares F (2008) A comparative study of the self-efficacy beliefs of successful men and women in mathematics, science, and technology careers. Journal of Research in Science Teaching 45:1036–1058.

[57] Berkman ND, Sheridan SL, Donahue KE, Halpern DJ, Viera A, et al**.** (2011) Health literacy interventions and outcomes: an updated systematic review. PMC4781058 23126607

[58] Annetta LA, Shymansky JA (2006) Investigating science learning for rural elementary school teachers in a professional-development project through three distance-education strategies. Journal of Research in Science Teaching 43:1019–1039.

[59] Davis EA, Krajcik JS (2005) Designing educative curriculum materials to promote teacher learning. Educational researcher 34:3–14.

