# Supplementary material for: Modeling for Fidelity: Virtual Mentorship by Scientists Fosters Teacher Self-Efficacy and Promotes Implementation of Novel High School Biomedical Curricula
Source: PLoS One. 2014 Dec 31;9(12):e114929. doi: 10.1371/journal.pone.0114929 (PMC4281152; doi:10.1371/journal.pone.0114929)
Supplement: S1 File — Supporting Figures and Tables providing extended datasets. (PDF) [file pone.0114929.s001.pdf]

Supplementary Figure A:

Heat map representing alignment of the Neuroscience module to the 3 dimensions of the NGSS.

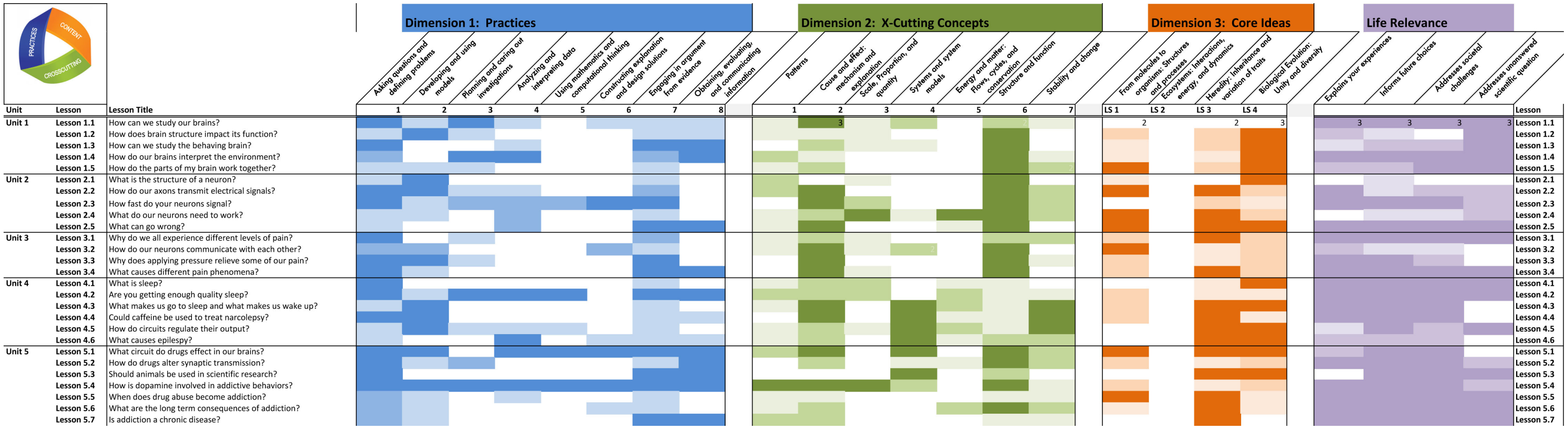

The NGSS defines three strands that are critical to the teaching of biology. To determine the extent to which each of the lessons in the module was aligned to each of the strands two Neuroscience content experts (KM and KFM) evaluated each of the lessons independently with respect to how well they aligned to the science practices (blue) cross cutting concepts (green) and core ideas (orange). The extent of alignment was color coded using a 1-3 scale with the darkest color equivalent to the highest degree of alignment. In addition, because our curriculum focuses heavily on the impact of science on health, we also developed a further dimension 'life relevance' (purple) which we aligned and color coded similarly.

Supplementary Table A. Demographics of classrooms for curriculum implementation.

	**Urban Exam**	**Urban General**	**Suburban General**	**Regional STEM**
**African American**	25	4	3	8
**Asian**	30	2	2	0
**Hispanic**	9	16	4	0
**Native American**	0	2	0	0
**White**	66	6	21	13
**Multi-race, non Hispanic**	5	0	0	0
**Enrollment**	**135**	**30**	**30**	**21**

Supplementary Table B: Study participants

	Enrolled	Conceptual knowledge	Attitude Self-efficacy
**Urban Exam**	135	117 (87%)	86 (64%)
**Urban General**	30	18 (60%)	20 (66%)
**Suburban General**	30	24 (80%)	24 (80%)
**Regional STEM**	21	16 (76%)	17 (81%)
**Total**	**212**	**175 (81%)**	**147 (68%)**

<u>**Supplementary Table C: Fidelity of Implementation**</u>

Critical Instructional/pedagogical components	Sample Question	Sample responses
**Facilitation of discussion**	Where there any questions your students had that you couldn't answer?	Students didn't understand and I didn't understand the difference between sleep and coma
**Facilitation of group work**	Did you adjust the group work in any way?	I had them do the manual demo of how neurons communicate first with actual students acting as sensory/pain neurons in a specific pathway.
**Facilitation of student interest**	Which lesson worked best for your students and why?	My students loved this lesson (3.2). The quiz, the videos, etc. It really was a great "hook" to the unit overall, and some difficult ideas ahead!
**Facilitation of students doing intellectually challenging work**	Which version of the lesson did you use?	2.2 (action potential) went very well! I was shocked at how much they seemed to grasp it when we first went through it (did the advanced one with sodium and potassium channels and the Na/K pump too). They were able to identify the ups and downs and what was happening when on tests too. I think this was because I asked them to make a lot of predictions and tell me what was going to happen before we discussed it. So they could see how it all made sense and had to explain why an influx of positive ions would cause the membrane potential to depolarize instead of just memorizing it. 3 (how fast neurons signal) went well too. It was a good activity and let them see how fast neurons really have to send signals.
**Facilitation of manipulatives**	How did the activity go?	This was a little tougher. The card activity was great and the visuals on the ppts were awesome. I had them do the manual demo of how neurons communicate first with actual students acting as sensory/pain neurons in a specific pathway. That seemed to drive home the card activity.
**Use of assessments**	How did you assess student mastery of the objectives from Unit 4?	Quiz over unit 4, formative assessments - homework, worksheets, in class questioning

Supplementary Table D: Online survey for Teachers

Survey Question	Sample response
What reservations, if any, did you have about using the support training?	I didn't have any reservations about the support training itself, but I was worried about the time commitment in addition to normal planning, lab prep, communication with parents, student support, etc.
What impacted your decision to use the support training?	I felt that the support training would be helpful in brushing up on content knowledge.
What did you find most useful about the support you received?	I think that the most useful aspect of the support was the regular access to an expert. If I encountered questions or extensions in my preparations or in my teaching, I always had an expert ready and eager to help.
What was most valuable about the support you received?	A heads-up about known issues/good things so nothing was ever a big shock when I did it in the classroom for the first time. When I was alerted to possible issues/difficulties, I did my best to try to modify things for my students. That was very helpful and I was generally successful. Also a "go to" person for questions! My students asked TONS of questions that I couldn't answer, so [having someone] able to answer questions was very nice!!!
What could be improved about the support you received?	If I could be in the same location as the support! Skype and Google Chat worked well, but I would have loved to be able to meet with someone in person, even if it was prior to or after implementation of the curriculum.
A scientist with extensive knowledge of the curriculum was the mentor. Do you think a person with this background is best suited for this role? Why or why not?	Yes -- absolutely. I believe that as a teacher, I have the expertise and ability to implement my curriculum with my students, however I sometimes feel that my content knowledge is lacking. The support that I find that I need as a teacher is support that deepens my understanding and extension of content. Rather that constantly working to try to re-learn or re-teach all the material to myself, it was incredibly helpful to have a mentor who I could rely on and interact with regularly. I didn't feel so "alone" in my classroom.
Do you have any additional comments, questions or concerns?	Really, just a sincere thanks. This is an amazing set up and offers a truly invaluable opportunity for teachers to grow in the classroom. It is professional development without the long, boring meetings!

<u>Supplementary Table E: Organization of the Neurological Disorders module into thematic units.</u>

Units	Unit theme and learning goals
**Unit 1: Got Brains: What do our brains need to do?**	*How do different parts of our brains function to create a coherent whole?* A sheep brain dissection introduces the concept of functional modules. Then students investigate how the brain sense the environment and establishes a complex sensory percept. Finally they investigate how different areas of the brain cooperate to control complex functions like language.
**Unit 2: What are the building blocks of our brains?**	*How is the brain put together and how do the individual building blocks function?* During this unit students learn how the neuron is put together, how it does what it does, and what can happen when different parts of the neuron fail to function. Students experience an engineering problem in which they are challenged to collaborate to design a faster, more efficient neuron.
**Unit 3: How do our neurons communicate with each other?**	*How are neurons are able to communicate with each other to send electrical signals from one part of the brain to another?* This unit uses our distinct perceptions of pain are formed by different parts of the brain collaborating to excite and inhibit each other.
**Unit 4: How do our neurons work together to control behaviors?**	*How do we know when to go to sleep and when to wake up?* This unit focuses on how the brain can control complex behaviors. Armed with their knowledge of how neurons can excite and inhibit each other students investigate how the sleep/wake circuit works, how sleep quality affects performance and how defects in neuronal circuits can lead to disrupts in function like epilepsy.
**Unit 5: How do our choices change our brains?**	*How can specific brain circuits drive our behaviors?* This unit focuses on the centrality of the reward pathway to a number of different addictive behaviors. Students draw on their knowledge of how neuronal circuits operate to investigate the drive to repeat destructive behaviors.
**Final project**	Students grapple with the idea of addiction as a neurological disorder and explore public policy issues of how to deal with substance abuse. They create a public service announcement explaining a particular addictive behavior in the context of brain function to communicate the levels of conscious and unconscious control the brain exerts.
